# Generalized cure rate model for infectious diseases with possible co-infections

**DOI:** 10.1371/journal.pone.0239003

**Published:** 2020-09-11

**Authors:** Oluwafemi Samson Balogun, Xiao-Zhi Gao, Emmanuel Teju Jolayemi, Sunday Adewale Olaleye

**Affiliations:** 1 School of Computing, University of Eastern Finland, Kuopio, Finland; 2 Department of Statistics, Faculty of Science, University of Ilorin, Ilorin, Kwara State, Nigeria; 3 Department of Marketing, Management and International Business, University of Oulu, Oulu, Finland; Tongii University, CHINA

## Abstract

This research mainly aims to develop a generalized cure rate model, estimate the proportion of cured patients and their survival rate, and identify the risk factors associated with infectious diseases. The generalized cure rate model is based on bounded cumulative hazard function, which is a non-mixture model, and is developed using a two-parameter Weibull distribution as the baseline distribution, to estimate the cure rate using maximum likelihood method and real data with R and STATA software. The results showed that the cure rate of tuberculosis (TB) patients was 26.3%, which was higher than that of TB patients coinfected with human immunodeficiency virus (HIV; 23.1%). The non-parametric median survival time of TB patients was 51 months, while that of TB patients co-infected with HIV was 33 months. Moreover, no risk factors were associated with TB patients co-infected with HIV, while age was a significant risk factor for TB patients among the suspected risk factors considered. Furthermore, the bounded cumulative hazard function was extended to accommodate infectious diseases with co-infections by deriving an appropriate probability density function, determining the distribution, and using real data. Governments and related health authorities are also encouraged to take appropriate actions to combat infectious diseases with possible co-infections.

## 1. Introduction

An infectious disease occurs when a disease agent invades a host and harm the host's tissues (i.e., they cause disease). These diseases can be transmitted to other individuals (i.e., they are infectious). There are five major types of infectious agents: bacteria, viruses, fungi, protozoa, and helminths [1]. Further, a new class of infectious agents, prions, has recently been discovered [1]. Tuberculosis (TB) is highly prevalent in Nigeria; hence, the Nigerian government has proclaimed that its treatment is free. However, the successes recorded in TB management have drastically reduced [2]. TB is a potentially severe infectious disease that mainly affects the lungs. The bacteria that cause TB spread from one individual to another through tiny droplets released into the air via coughs and sneezes. TB is caused by the bacteria known as *Mycobacterium tuberculosis* and is curable and preventable. According to the World Health Organization, approximately one-quarter of the world's population has latent TB, in which infected people show no symptoms of the disease and cannot transmit it [3, 4]. People infected with *M*. *tuberculosis* have a 5–15% lifetime risk of falling ill with TB. In any case, persons with compromised immune systems, such as people with human immunodeficiency virus (HIV), malnutrition, diabetes, or people who use tobacco, have a much higher risk of falling ill when they develop active TB disease [3, 4].

An efficient alternative model to standard Cox proportional hazard models [5] for data with trends in survival like those shown in Fig 1 of [6], on several grounds is cure models. First, when the survival curves plateau is at their tails, the assumption of proportional hazards can fail. Second, long plateau survival plots may indicate heterogeneity within the patient population that may be useful in the data's explicit description. Cure models allow us to examine covariates that are either associated with short-term or long-term effects. They also allow us to assess whether new therapy increases or decreases the likelihood that the patient will be permanently cured, respond to treatment, or die [5]. Meanwhile, the cure fraction model indicates the fraction of patients who survive any disease for an extended period. Cure models center on the probability of the survival of an uncured patient up to a given point in time [7].

Recently, the use of cure models for examining single diseases has become increasingly popular. However, to the authors’ knowledge, the application of cure rate model to infectious diseases with possible co-infections has scarcely been investigated. Hence, a real dataset of TB patients co-infected with HIV was used in this work to develop a generalized cure rate model for estimating the number of patients who are cured, estimate their survival rate, and identify the risk factors associated with the diseases. Several authors [8–13] have used bounded cumulative hazard model with the Expectation-Maximization algorithm, as well as the maximum likelihood estimation, for the cure rate model to estimate the cure rate of single diseases. The generalized cure rate model proposed in this research is based on two-parameter Weibull distribution, which is an extension of the BCH model, a non-mixture model. The remainder of this paper is organized as follows: in Section 2, past studies are reviewed; in Section 3, the methods used and ethical approval are discussed; in Section 4, the analysis and results are presented; the results are discussed in Section 5; and conclusion and recommendations are presented in Section 6.

## 2. Literature review

The symptoms of TB include cough, fever, weight loss, or night sweats, and may be mild for several months. These mild symptoms can lead to delays in seeking medical attention, thereby resulting in the transmission of the bacteria to other individuals. People with active TB can infect 150 other individuals through close contact over a year. Without proper treatment, an average of 45% of HIV-negative patients with TB and almost all HIV-positive patients with TB will die [3, 4].

Several research and development efforts are ongoing to improve the lifespan of patients experiencing a wide range of deadly diseases, including cancer, TB, and HIV/AIDS. Most patients experiencing a specific type of cancer have been permanently cured. A large proportion of patients who respond positively to treatment are usually free of any symptoms of the disease and regarded as cured, long-term survivors, or immune against the disease. Meanwhile, other patients who do not respond to treatment or relapse are considered to be susceptible to the disease or uncured.

Varshney et al. [14] built a cure model using a real HIV/AIDS survival dataset under a Bayesian setup to improve the applicability of cure models based on exponential, generalized exponential, Raleigh, Weibull, and exponentiated Weibull distributions. Meanwhile, Shi and Yin [15] proposed a landmark cure rate model that incorporates a time-dependent covariate to obtain dynamic predictions of a patient's survival possibilities as new clinical information emerges during follow-up. The model was based on the Cox proportional hazard, accelerated failure time, and censored quantile regression models in the presence of a cure proportion. Simulation and real-life data were then used to assess the accuracy of the proposed method in the work done by Shi and Yin. However, a new survival model that assumes the semiparametric Bayesian approach is proposed in this study by imposing a Gaussian process before the nonlinear structure of continuous covariates. This development enables the right-censored survival data of patients to be analyzed if the log failure time follows a generalized extreme value distribution, using simulated data and real dataset [16]. In addition, a non-mixture non-parametric cure rate model was applied to real data after using stepwise selection to determine the risk factors for colorectal cancer [17].

This research combined the cure fraction model based on generalized modified Weibull distribution and the inferences obtained using Markov chain Monte Carlo method, which is a Bayesian approach for determining the risk factors associated with breast cancer [18].

Moreover, the proposed survival cure rate model was established by modeling a few concurrent causes using the Yule-Simon distribution. The model parameters were obtained using maximum likelihood estimation. Furthermore, a real dataset was used to show that the proposed model outperforms traditional alternative models in terms of model fitting [19]. Sun et al. [20] proposed some safety factors for traffic congestion in China based on accident risk and prevention. Additionally, Zeng et al. [21] examined the risks associated with the crash rate based on severity. Thus, the aim of this study is to examine the risk factors associated with TB [22, 23].

Some researchers [24–26] have applied the cure rate model to evaluate loan performance and determine loan recovery. Additionally, the model has been applied to determine the percentage of convicts that would return to jail [27].

In this study, a flexible cure model was proposed using survival data with a power series distribution, and Bernoulli and geometric Poisson distributions were generalized to determine the best fit using cutaneous melanoma data [28].

## 3. Material and methods

Secondary data from 2000 to 2015 obtained from the University of Ilorin Teaching Hospital (UITH), Nigeria were used for this work.

The data comprised 518 observations of TB patients with age (in years) and gender (male = 1, female = 0), as well as the time taken (in months) for each patient to be cured. Moreover, the data comprised 133 observations of TB patients co-infected with HIV with age (in years), gender (male = 1, female = 0), and the time taken (in months) for each patient to be cured. Although different types of data can be used for this research, such as prevalence and hospital data, hospital data are preferable because the patients' data are collected from the medical department of a government-approved hospital. The model used in this work was applied to only single infectious diseases in past studies. In this study, the model was modified into a generalized cure rate model, which can be applied to any infectious disease with possible co-infections. Data analysis was performed using STATA, SPSS, and "Model adequacy", which is a package in R software [29].

### 3.1. Standard cure rate model

The standard cure rate model, which is the foundation of the proposed model, was modified and its parameters are defined as follows: *π* is the probability of a patient with an infectious disease and a possible co-infection being a long-term survivor, and (1−*π*) is the probability of a patient being susceptible. Therefore, the entire population survival function at any time t is given as:

$$S(t)=\pi +(1-\pi )S_{u}(t)$$ (1)

where *S*_*u*_(*t*) is the survival function of the susceptible population, which may be assumed to follow a lifetime distribution. Meanwhile, exponential, gamma, Weibull, Rayleigh, generalized exponential, and exponentiated Weibull distribution can be used to estimate the cure fraction, *π*. The probability density function, *f*(*t*), of the overall population is given as:

$$f(t)=(1-\pi )f_{u}(t)$$ (2)

where is *f*_*u*_(*t*) is the probability density function of the susceptible population.

### 3.2. Assumptions of the bounded cumulative hazard model

1. The model originated through a natural biological motivation.
2. The model is readily calculable because the cure rate parameter allows for the existence of a proportional hazard structure.
3. The model enables an efficient posterior distribution sampling of the parameters by proposing a latent variable that makes it computationally beneficial.
4. The proposed cure rate model can be considered as a standard, revealing the mathematical relationship between the standard cure rate and the model.

### 3.3. Generalized cure rate model

In this study, a modified model was developed using bounded cumulative hazard function, a non-mixture model. The modified model is referred to as generalized cure rate model with survival function, and is given as [30]:

$$S(t)=\underset{\left(1\right)}{\underbrace{{\left[\pi +(1-\pi )S_{u}(t)\right]}^{1-d_{i}}}}\times \underset{\left(2\right)}{\underbrace{{\left[\pi +(1-\pi )S_{u}(t)\right]}^{d_{i}}}},$$ (3)

where *π* = *exp*(−*θ*) and 1-*π* = 1−*exp*(−*θ*). Here, *θ* is the mean number of occurrences of the disease. In Eq (3), Eq (1) is used for patients with single infectious diseases and Eq (2) is used for patients with co-infected diseases. Note that Eq (3) is an extension of Eq (1), and it can be applied for single diseases with possible co-infections when a new parameter, d_i_, which denotes the type of disease, is introduced.

Assuming that (*α*_*i*_, *c*_*i*_, *t*_*i*_, *d*_*i*_) is the observed data of size n, where *t*_*i*_ is the survival time of the ith patient; *α*_*i*_ denotes the censoring indicator variable, where *α*_*i*_ = 0 for an uncensored observation and *α*_*i*_ = 1 for a censored observation; *c*_*i*_ denotes the cure indicator variable, where *c*_*i*_ = 0 for a cured patient and *c*_*i*_ = 1 for an uncured patient; and *d*_*i*_ denotes the disease indicator variable, where *d*_*i*_ = 0 for a single infectious disease and *d*_*i*_ = 1 for co-infected diseases (i = 1, 2,.., n).

The individual patient’s contribution to the likelihood function is given by

$$l_{c}=\left\{\begin{array}{l} {\left[\log\prod_{i=1}^{n}{\left[\{f_{u}\left(t_{i}\right){\left(1-\pi \right)\}}^{c_{i}}\right]}^{\alpha_{i}}{\left[{\left\{\pi \right\}}^{1-c_{i}}{\left\{\left(1-\pi \right)\left(1-S_{u}\left(t\right)\right)\right\}}^{c_{i}}\right]}^{1-\alpha_{i}}\right]}^{1-d_{i}}\times \\ \;{\left[\log\prod_{i=1}^{n}{\left[\{f_{u}\left(t_{i}\right){\left(1-\pi \right)\}}^{c_{i}}\right]}^{\alpha_{i}}{\left[{\left\{\pi \right\}}^{1-c_{i}}{\left\{\left(1-\pi \right)\left(1-S_{u}\left(t\right)\right)\right\}}^{c_{i}}\right]}^{1-\alpha_{i}}\right]}^{d_{i}} \end{array}\right\}$$ (4)

Using exponential distribution as the baseline distribution for *S*_*u*_(*t*), *f*_*u*_(*t*) becomes:

$$f_{u}\left(t_{i}\right)=\frac{k}{\beta^{k}}t^{k-1}\exp\left(-{\left(\frac{t}{\beta }\right)}^{k}\right)\;S_{u}\left(t_{i}\right)=\exp\left(-{\left(\frac{t}{\beta }\right)}^{k}\right)$$ (5)

Hence, the complete data likelihood is given by:

$$l_{c}=\left\{\begin{array}{l} {\left[\log\prod_{k=1}^{n}{\left[{\left\{\frac{k}{\beta^{k}}t^{k-1}\exp\left(-{\left(\frac{t}{\beta }\right)}^{k}\right)\left(1-e^{-\theta }\right)\right\}}^{c_{i}}\right]}^{{}^{\alpha_{i}}}{\left[{\left\{e^{-\theta }\right\}}^{1-c_{i}}{\left\{\left(1-e^{-\theta }\right)\left(1-\exp\left(-{\left(\frac{t}{\beta }\right)}^{k}\right)\right)\right\}}^{c_{i}}\right]}^{1-\alpha_{i}}\right]}^{1-d_{i}}\\ \times {\left[\log\prod_{k=1}^{n}{\left[{\left\{\frac{k}{\beta^{k}}t^{k-1}\exp\left(-{\left(\frac{t}{\beta }\right)}^{k}\right)\left(1-e^{-\theta }\right)\right\}}^{c_{i}}\right]}^{{}^{\alpha_{i}}}{\left[{\left\{e^{-\theta }\right\}}^{1-c_{i}}{\left\{\left(1-e^{-\theta }\right)\left(1-\exp\left(-{\left(\frac{t}{\beta }\right)}^{k}\right)\right)\right\}}^{c_{i}}\right]}^{1-\alpha_{i}}\right]}^{d_{i}} \end{array}\right\}$$ (6)

Simplifying Eq (6):

$$\begin{array}{c} l_{c}=\sum_{i=1}^{m_{1}}\left[(1-d_{i})c_{i}\alpha_{i}][\log\;k-[k\;\log\;\beta ]+[(k-1)\log\;t]-{\left(\frac{t}{\beta }\right)}^{k}+\log\left(1-e^{-\theta }\right)\right]+\\ \sum_{i=1}^{m_{1}}\left[(1-d_{i}\right)(1-\alpha_{i})]\left[[(1-c_{i})(-\theta )]+c_{i}\{[\log\left(1-e^{-\theta }\right)]+\log\left(1-\exp\left(-{\left(\frac{t}{\beta }\right)}^{k}\right)\right)\}\right]\\ +[\sum_{i=1}^{m_{2}}\left(d_{i})c_{i}\alpha_{i}][\log\;k-[k\;\log\;\beta ]+[(k-1)\log\;t]-{\left(\frac{t}{\beta }\right)}^{k}+\log\left(1-e^{-\theta }\right)\right]+\\ \sum_{i=1}^{m_{2}}\left[\left[(d_{i})(1-\alpha_{i})][\left[(1-c_{i})(-\theta )]+c_{i}\{[\log\left(1-e^{-\theta }\right)]+\log\left(1-\exp\left(-{\left(\frac{t}{\beta }\right)}^{k}\right)\right)\right\}\right]\right] \end{array}$$ (7)

The solutions of $\frac{\partial l_{c}}{\partial \theta },\frac{\partial l_{c}}{\partial k},$ and $\frac{\partial l_{c}}{\partial \beta }=0$ are the desired estimates of *θ*, *k*, and *β*, where

$$\frac{\partial l_{c}}{\partial \theta }=\frac{\sum_{i=1}^{m_{1}}(1-d_{i})c_{i}}{\left(1-e^{-\theta }\right)}+\frac{\sum_{i=1}^{m_{2}}d_{i}c_{i}}{\left(1-e^{-\theta }\right)}-[\sum_{i=1}^{m_{1}}[(1-d_{i})(1-\alpha_{i})(1-c_{i})]+\sum_{i=1}^{m_{2}}\left[(d_{i})(1-\alpha_{i})(1-c_{i})\right]]$$ (8)

$$\begin{array}{l} \;\frac{\partial l_{c}}{\partial k}=\frac{\sum_{i=1}^{m_{1}}(1-d_{i})c_{i}\alpha_{i}}{k}-\sum_{i=1}^{m_{1}}\left[(1-d_{i}\right)c_{i}\alpha_{i}\;\log\;\beta ]+\sum_{i=1}^{m_{1}}\left[(1-d_{i}\right)c_{i}\alpha_{i}\;\log\;t]\\ \;-\sum_{i=1}^{m_{1}}\left[(1-d_{i})c_{i}\alpha_{i}\;\log\left(\frac{t}{\beta }\right){\left(\frac{t}{\beta }\right)}^{k}\right]+\\ \;\sum_{i=1}^{m_{1}}\left[(1-d_{i})(1-\alpha_{i})\log\left(\frac{t}{\beta }\right)\frac{\log\;e}{e^{{\left(\frac{t}{\beta }\right)}^{k}}-1}{\left(\frac{t}{\beta }\right)}^{k}\right]+\frac{\sum_{i=1}^{m_{2}}d_{i}c_{i}\alpha_{i}}{k}\\ \;-\sum_{i=1}^{m_{2}}[d_{i}c_{i}\alpha_{i}\;\log\;\beta ]+\sum_{i=1}^{m_{2}}[d_{i}c_{i}\alpha_{i}\;\log\;t]-\\ \sum_{i=1}^{m_{2}}\left[d_{i}c_{i}\alpha_{i}\;\log\left(\frac{t}{\beta }\right){\left(\frac{t}{\beta }\right)}^{k}\right]+\sum_{i=1}^{m_{2}}\left[d_{i}(1-\alpha_{i})\log\left(\frac{t}{\beta }\right)\frac{\log\;e}{e^{{\left(\frac{t}{\beta }\right)}^{k}}-1}{\left(\frac{t}{\beta }\right)}^{k}\right] \end{array}$$ (9)

$$\begin{array}{l} \frac{\partial l_{c}}{\partial \beta }=-\frac{\sum_{i=1}^{m_{1}}(1-d_{i})c_{i}\alpha_{i}k}{\beta }-\sum_{i=1}^{m_{1}}\left[\left(1-d_{i})c_{i}\alpha_{i}{\left(\frac{t}{\beta }\right)}^{k}(\frac{k}{\beta }\right)\right]+\sum_{i=1}^{m_{1}}\left[(1-d_{i})(1-\alpha_{i})k\right]\left[\frac{\log\;e}{\beta -\beta e^{{\left(\frac{t}{\beta }\right)}^{k}}}{\left(\frac{t}{\beta }\right)}^{k}\right]\\ -\frac{\sum_{i=1}^{m_{2}}d_{1}c_{i}\alpha_{i}k}{\beta }-\sum_{i=1}^{m_{2}}\left[d_{i}c_{i}\alpha_{i}{\left(\frac{t}{\beta }\right)}^{k}(\frac{k}{\beta })\right]+\sum_{i=1}^{m_{2}}\left[d_{i}(1-\alpha_{i})k][\frac{\log e}{\beta -\beta e^{{\left(\frac{t}{\beta }\right)}^{k}}}{\left(\frac{t}{\beta }\right)}^{k}\right] \end{array}$$ (10)

Solving Eq (8), we obtain:

$$\theta =\log\left[\frac{\sum_{i=1}^{m_{1}}\left(1-d_{i}\right)c_{i}+\sum_{i=1}^{m_{2}}d_{i}c_{i}}{\sum_{i=1}^{m_{1}}[\left(1-d_{i}\right)\left(1-\alpha_{i}\right)\left(1-c_{i}\right)]+\sum_{i=1}^{m_{2}}\left[\left(d_{i}\right)\left(1-\alpha_{i}\right)\left(1-c_{i}\right)\right]}+1\right]$$ (11)

Note that *m*_1_ is the sample of patients with a single disease and *m*_2_ is the sample of patients with co-infected diseases.

### 3.4. Estimation of model parameters for the Weibull distribution

Maximum likelihood estimation is useful for estimating the parameters of a two-parameter Weibull distribution because it is easy to compute the parameters of the distribution and a package exists for the distribution in R software.

### 3.5 Two-parameter Weibull distribution

$$f\left(t\right)=\frac{k}{\beta^{k}}t^{k-1}e^{-{\left(\frac{t_{i}}{\beta }\right)}^{k}},k>0,\beta >0$$ (12)

The likelihood function is given as:

$$\begin{array}{c} InL_{c}=\sum_{i=1}^{n}\left[In\left(k\right)-kIn\left(\beta \right)+\left(k-1\right)Int_{i}-{\left(\frac{t_{i}}{\beta }\right)}^{k}\right]\\ L_{c}=nIn\left(k\right)-nkIn\left(\beta \right)+\sum_{k=1}^{n}\left(k-1\right)Int-\sum_{k=1}^{n}{\left(\frac{t}{\beta }\right)}^{k} \end{array}$$ (13)

The values of the parameters of the log maximum likelihood estimate for the distribution for the two situations (TB alone and TB co-infected with HIV) were computed using an R code specifically written for this purpose.

### 3.6 Ethical considerations

The Ethical Research Committee of UITH approved the study according to Decision No. NHERC/02/05/2010 dated October 26, 2015. A written consent given for the data collection. The research team obtained approval to conduct the study from the hospital board before undertaking data collection. Data were obtained from the medical department of UITH, Ilorin, Kwara State, from 2000 to 2015 for TB patients and TB patients co-infected with HIV. All personal information related to the patients were anonymized. The study results will be disseminated to relevant stakeholders to inform policies and interventions to improve patient health and pave the way for future studies.

## 4. Results

In this section, the data used and the results of the analysis are presented. The dataset comprised observations for 518 TB patients with 298 males and 220 females, and 133 TB patients co-infected with HIV with 53 males and 80 females.

## 5. Discussion

**Fig 1:**
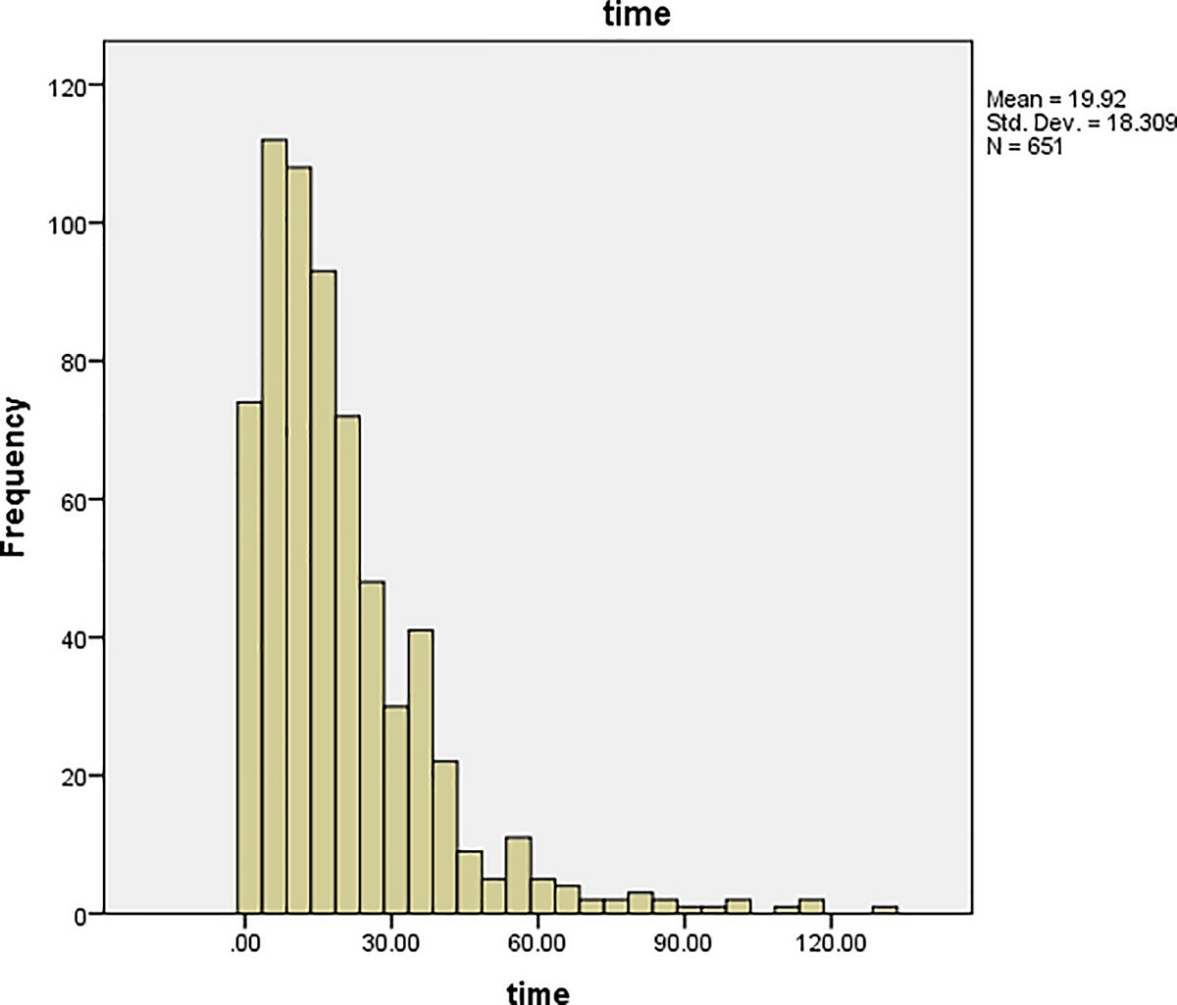
Graph of time for all the patients.

**Fig 2:**
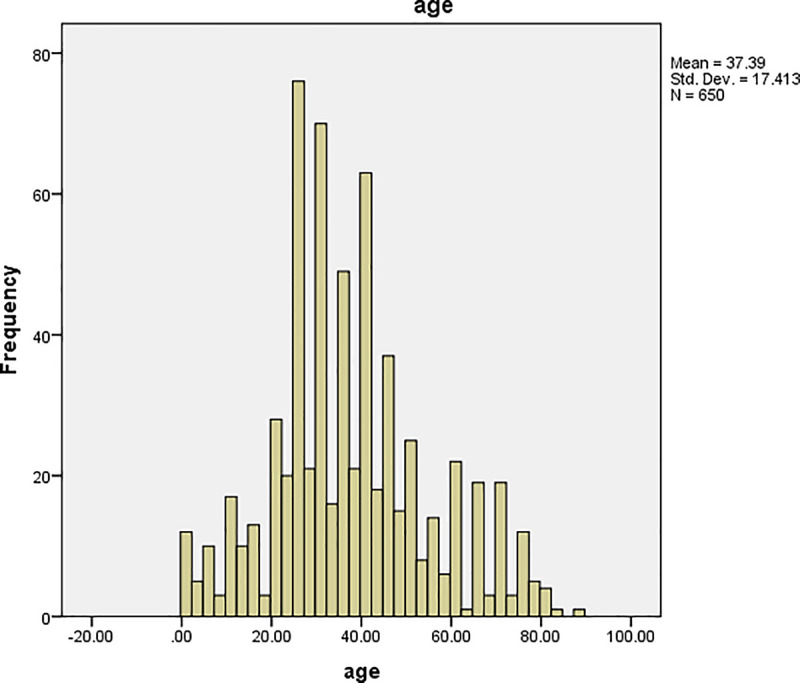
Graph of age for all the patients.

**Fig 3:**
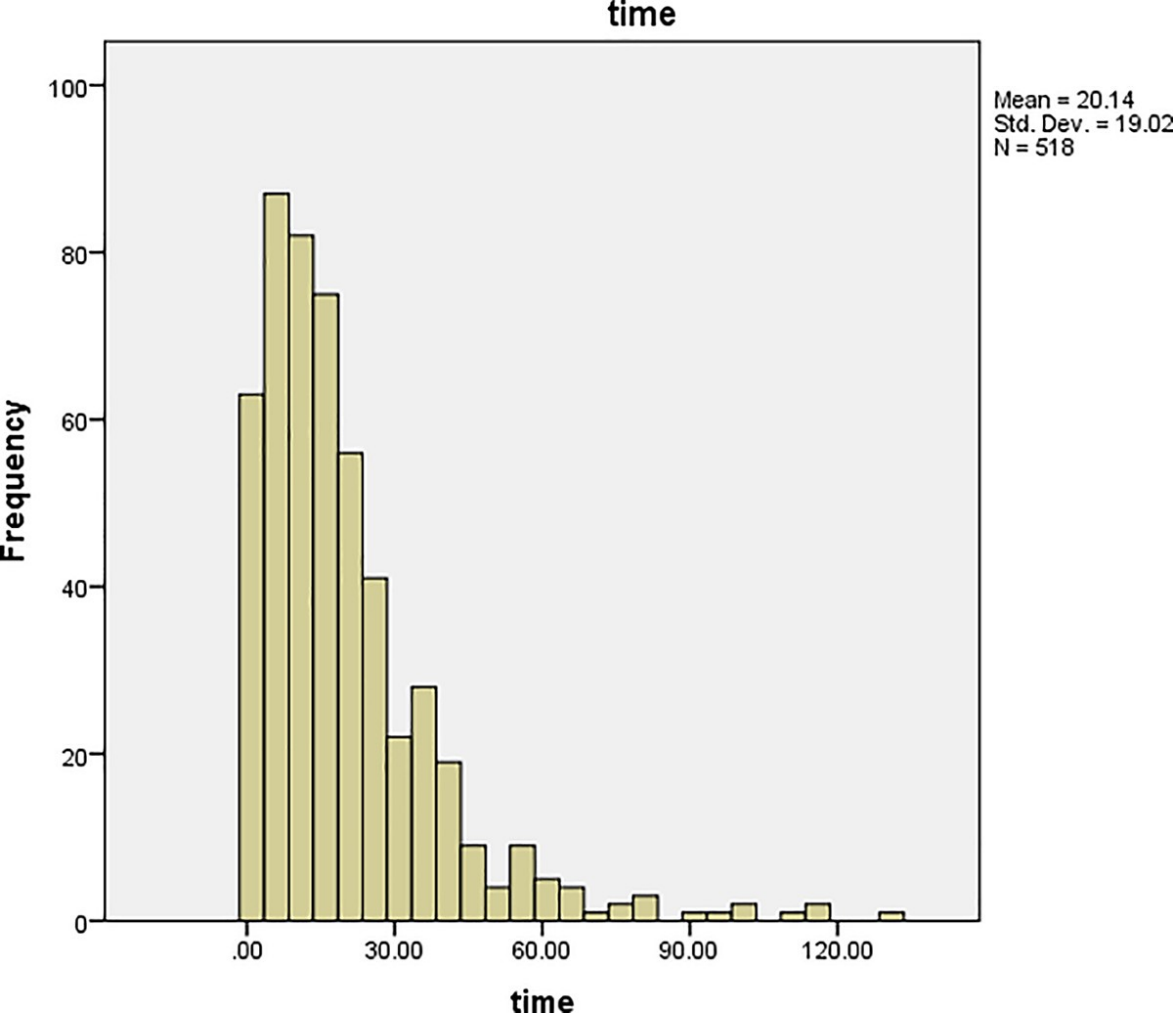
Graph of time for TB patients.

**Fig 4:**
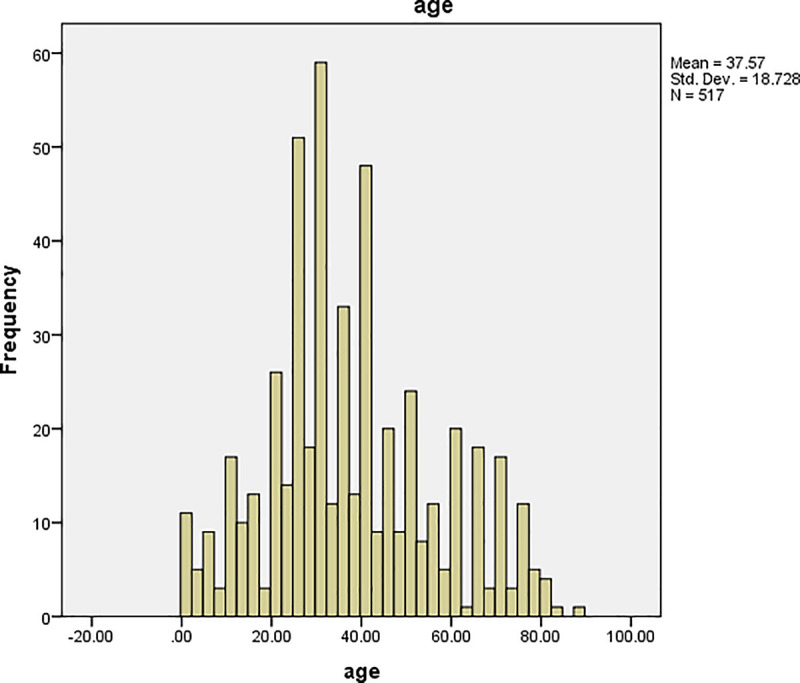
Graph of age for TB patients.

**Fig 5:**
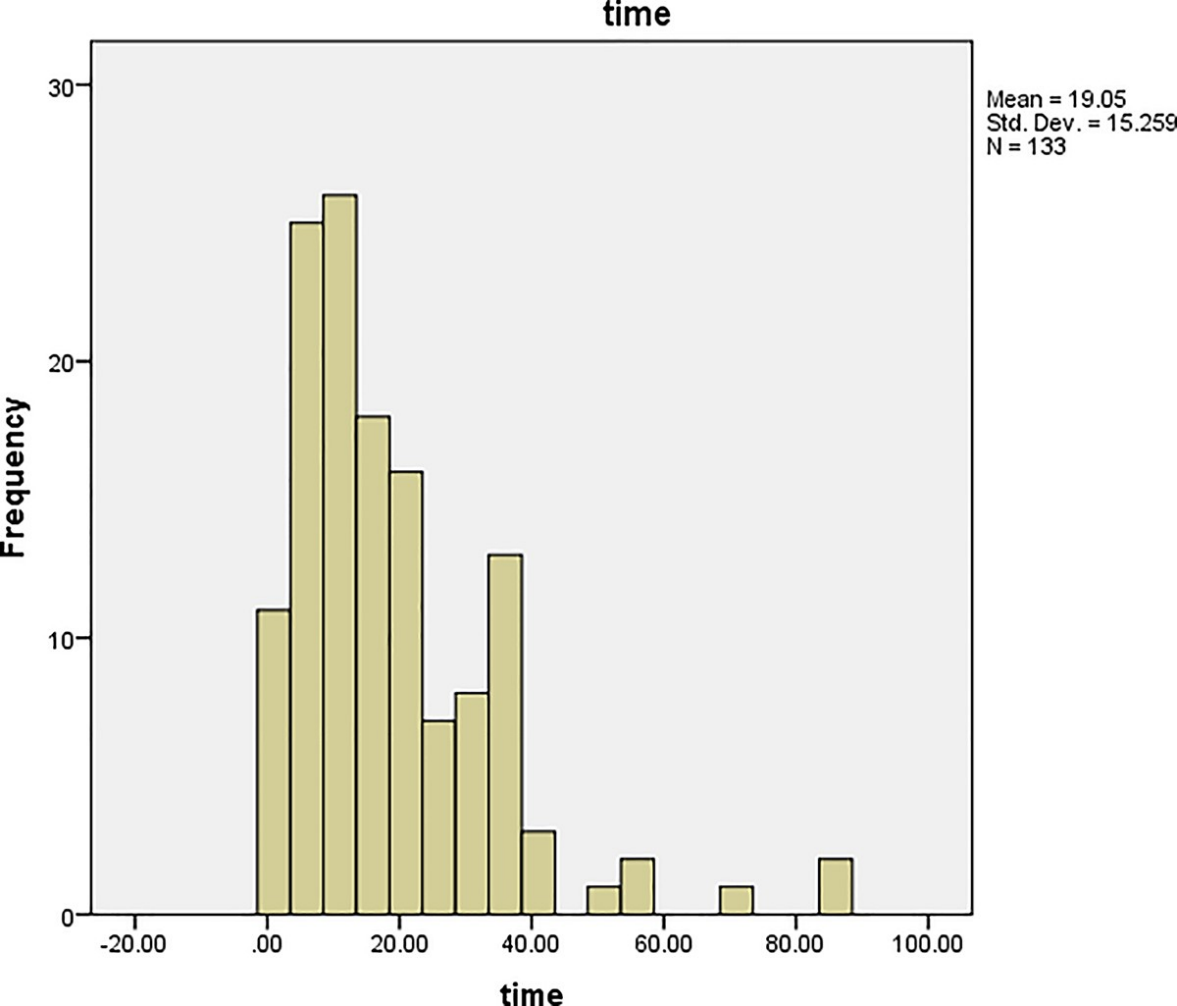
Graph of time for TB patients co-infected with HIV.

**Fig 6:**
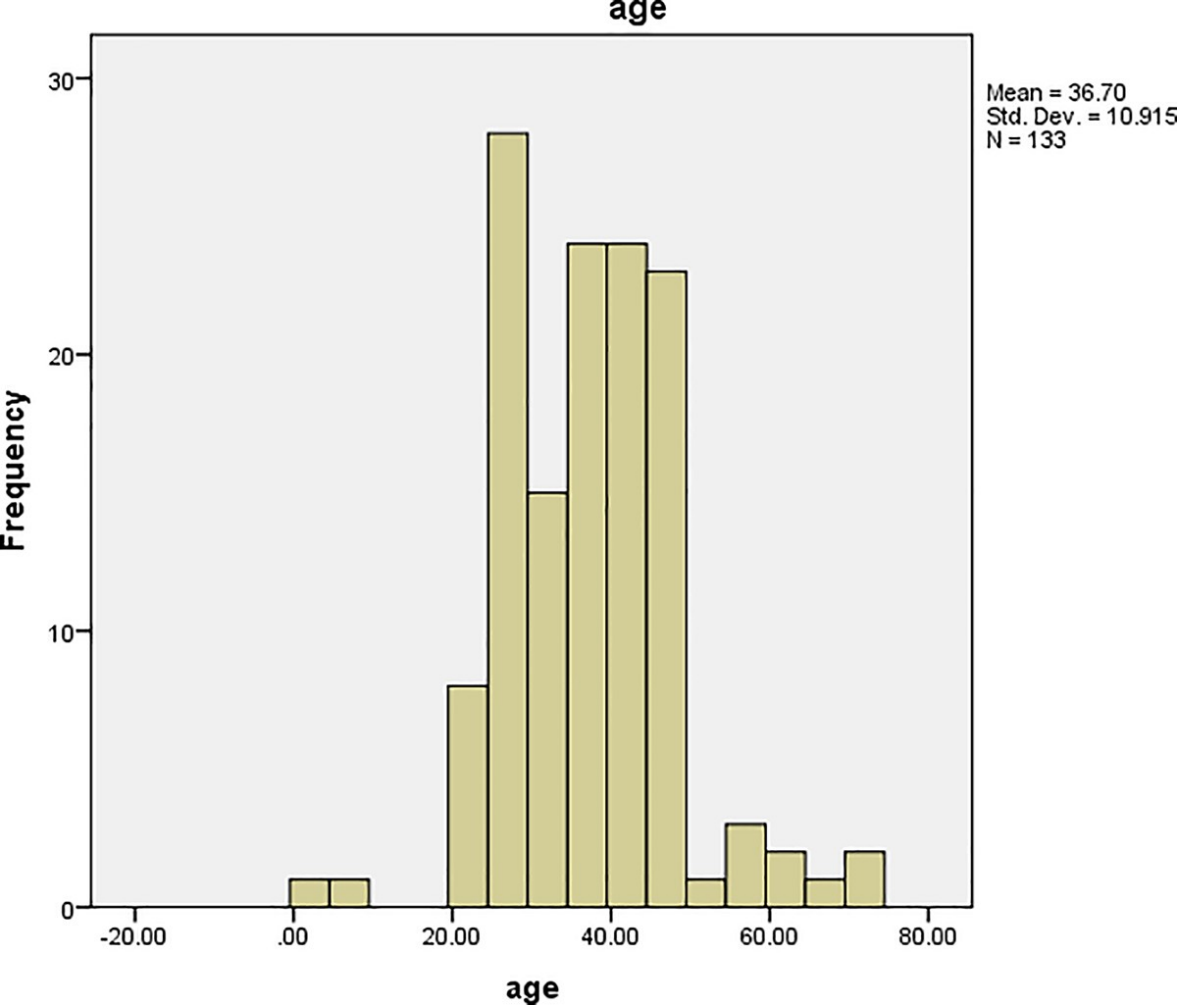
Graph of age for TB patients co-infected with HIV.

The graphs of time and age for all the patients are shown in Figs 1 and 2, respectively, and the corresponding graphs for TB patients are shown in Figs 3 and 4. Furthermore, the graphs of time and age for TB patients co-infected with HIV are shown in Figs 5 and 6, respectively. The results imply that age and time (length of stay of patients) are probable risk factors of TB and TB co-infection with HIV.

**Fig 7:**
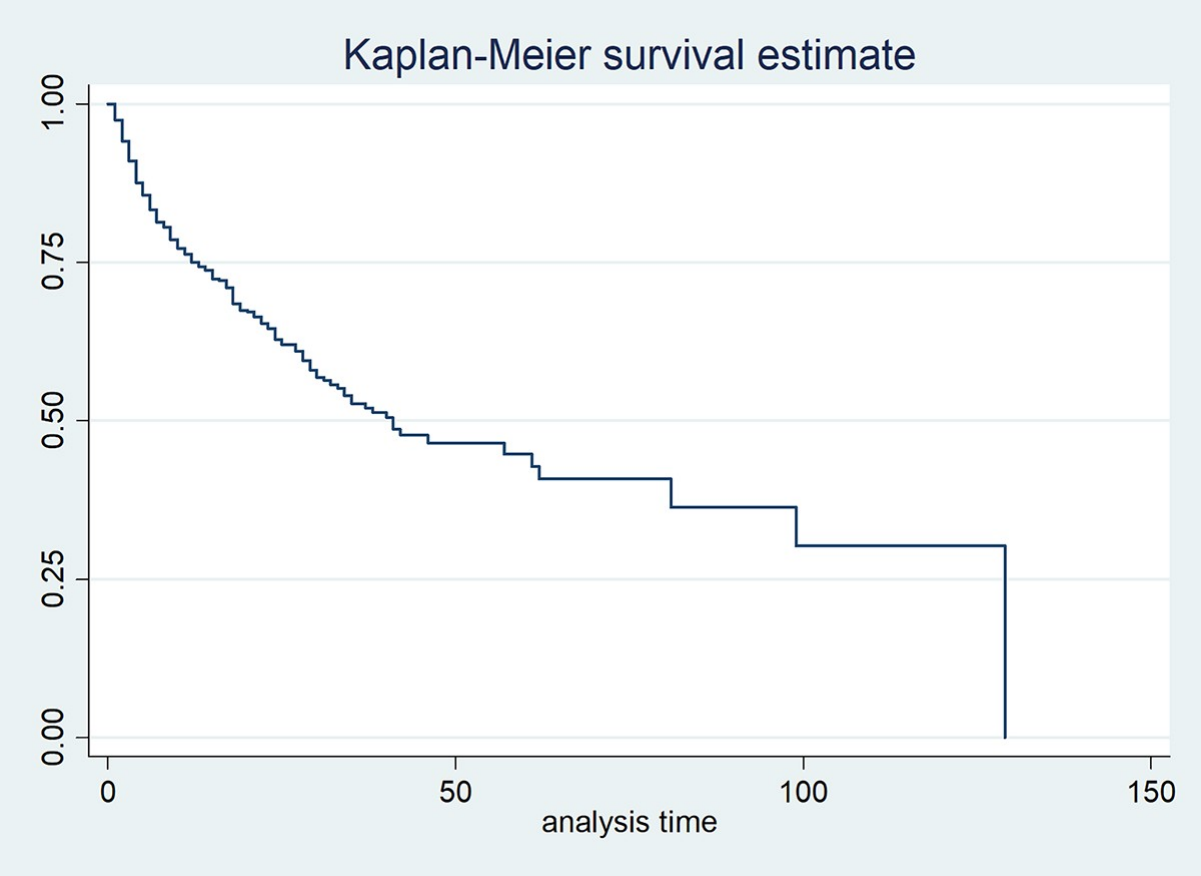
Graph of Kaplan-Meier survival estimate for TB patients.

From Table 1, the median, minimum, and maximum follow-up time of TB patients are 15, 1, and 129 months, respectively, whereas the corresponding follow-up time for TB patients co-infected with HIV are 15, 1, and 88 months, respectively. Since the median follow-up time of the two cases is the same, the management is independent of co-infection or not. Meanwhile, from Table 2 and Fig 7, the median, minimum, and maximum survival time of TB patients are 41, 13, and 129 months, respectively. The specified survival time signifies how long it takes to monitor TB patients irrespective of their gender.

**Table 1:** Follow-up time for all the patients.

Disease	Total	Follow-up time			Mean
		Min.	Median	Max.	
TB	518	1	15	129	20.12355
TB coinfected with HIV	133	1	15	88	19.05263

**Table 2:** Survival time for TB patients.

Gender	No. of subjects	Survival time		
		Min.	Median	Max.
Female	290	15	38	Not Available
Male	295	11	42	129
Total	517	13	41	129

**Fig 8:**
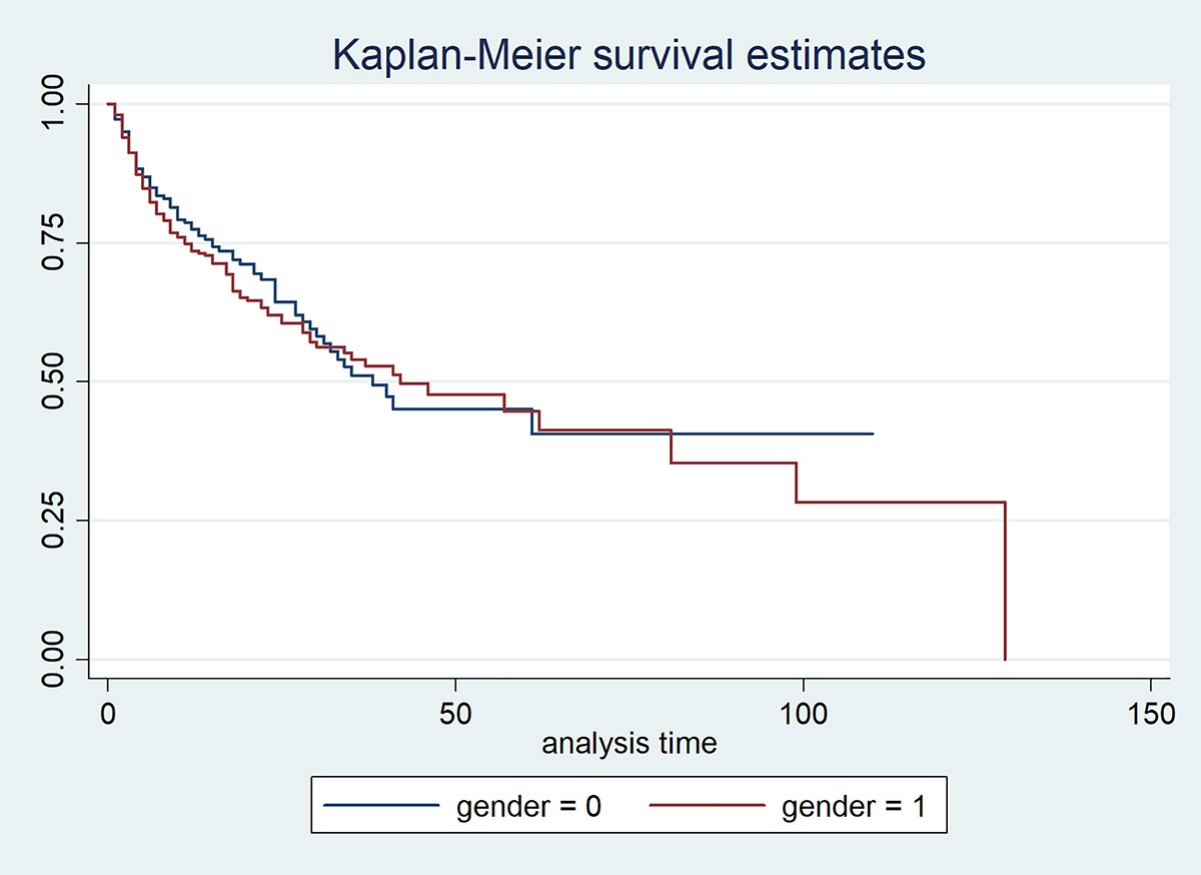
Graph of Kaplan-Meier survival estimates for TB patients based on gender.

Furthermore, the median, minimum, and maximum survival time of male TB patients are 42, 11, and 129 months, respectively (Table 2, Fig 8), whereas the median and minimum survival time of female TB patients are 38 and 15 months, respectively (Table 2, Fig 8). The survival time indicates how long it takes for both genders of TB patients to be monitored.

**Fig 9:**
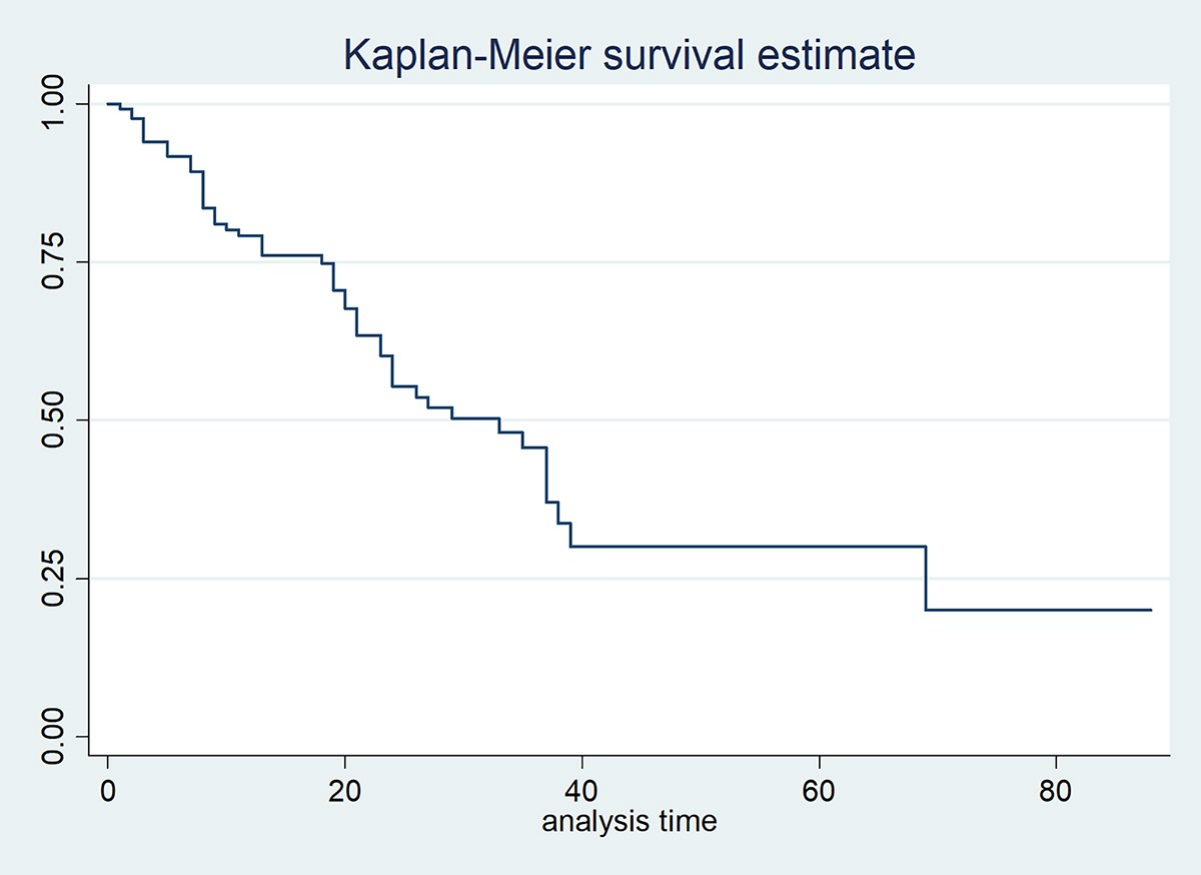
Graph of Kaplan-Meier survival estimate for TB patients co-infected with HIV.

Similarly, from Table 3 and Fig 9, the median, minimum, and maximum survival time of TB patients co-infected with HIV are 33, 18, and 69 months, respectively, indicating how long it takes for TB patients co-infected with HIV to be monitored irrespective of their gender.

**Table 3:** Survival time for TB patients co-infected with HIV.

Gender	No. of subjects	Survival time		
		Min.	Median	Max.
Female	80	19	37	39
Male	53	18	24	Not available
Total	133	18	33	69

**Fig 10:**
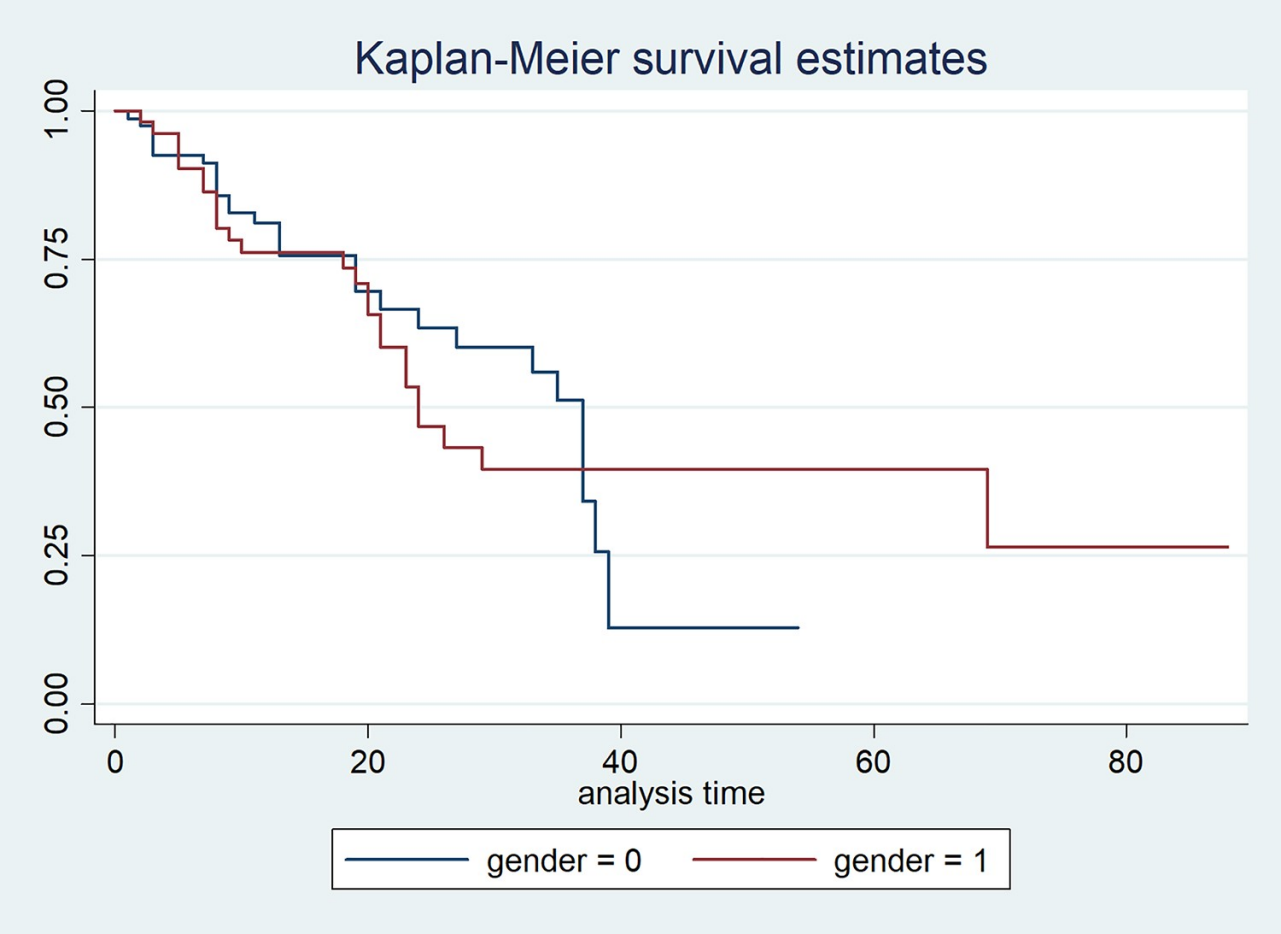
Graph of Kaplan Meier survival estimates for TB patients co-infected with HIV based on gender.

Moreover, the median, minimum, and maximum survival time of female TB patients co-infected with HIV are 37, 19, and 39 months, respectively (Table 3, Fig 10), whereas the minimum and maximum survival time of male TB patients co-infected with HIV are 18 and 24 months, respectively (Table 3, Fig 10), indicating how long it takes for both genders to be monitored.

The parameters of the two-parameter Weibull distribution—KS, P-value, AIC, and LLF—were obtained for both TB patients and TB patients co-infected with HIV using the “Model Adequacy” package of R software.

From Table 4, the p-value of the two-parameter Weibull distribution is 47% (0.46770), indicating the proportion of cured and uncured TB patients, their variances, and bias.

**Table 4:** Model parameters for TB patients.

Distribution	Parameters			KS	P-value	AIC	-LLF
	*β*	*k*	*μ*				
Weibull (2P)	21.149293	1.136517		0.037281	0.46760	4137.018	2066.509
Disease	N	*π* (%)	(1−*π*) (%)	P-value	Bias	Variance	MSE
TB	518	26.3	73.7	0.0001	0.0006612	0.23405	0.25135

Moreover, from Table 5, the p-value of the two-parameter Weibull distribution is 49% (0.48880), indicating the proportion of cured and uncured TB patients co-infected with HIV, their variances, and bias.

**Table 5:** Model parameters for TB patients co-infected with HIV.

Distribution	Parameters			KS	P-value	AIC	-LLF
	*β*	*k*	*μ*				
Weibull (2P)	20.778402	1.339535		0.072378	0.48880	1035.659	515.8297
Disease	N	*π* (%)	(1−*π*) (%)	P-value	Bias	Variance	MSE
TB co-infection with HIV	133	23.1	76.9		0.0005798	0.35634	0.36965

The results show that the p-value of TB patients is lower than that of TB patients co-infected with HIV, which implies that TB patients are cured faster than TB patients co-infected with HIV.

The Cox proportional hazard regression results are given in Table 6, in which the hazard ratio for TB patients increases with age and gender. Female patients have a higher risk than male patients. As the p-value for age (0.000) is less than 0.05 (*α*), age is a significant risk factor for the disease.

**Table 6:** Cox proportional hazard regression for TB patients.

T	Hazard ratio	Standard error	*Z*	*p*>⌈*z*⌉	95% *Conf*. *Interval*	
Age	1.020326	0.0038047	5.40	0.000	1.012896	1.02781
Gender	1.035359	0.1535419	0.23	0.815	0.7742109	1.384594
Constant	0.0127696	0.0033331	-16.71	0.000	0.0076559	0.021299

In addition, the hazard ratio for TB patients co-infected with HIV increases with age and decreases with gender, as shown in Table 7. Male patients have a lower risk than female patients. However, the p-values for age (0.168) and gender (0.700) are greater than 0.05 (*α*). Therefore, age and gender are not significant risk factors for the disease.

**Table 7:** Cox proportional hazard regression for TB patients co-infected with HIV.

T	Hazard ratio	Standard error	*Z*	*p*>⌈*z*⌉	95% *Conf*. *Interval*	
Age	1.017862	0.0130652	1.38	0.168	0.9925743	1.043794
Gender	0.8985374	0.2498049	-0.38	0.700	0.5210642	1.549463
Constant	0.0055466	0.0039964	-7.21	0.000	0.0013512	0.0227683

From the data observation, 26.3% of TB patients were cured, whereas 73.7% were uncured (Table 4). Besides, 23.1% of TB patients co-infected with HIV were cured, whereas 76.9% were uncured, which was alarming (Table 5). The result indicates that the proportion of TB patients who were cured is significantly higher (*p* < .0001) than that of TB patients co-infected with HIV.

In addition, TB patients respond well to the treatment received in the hospital compared to TB patients co-infected with HIV. The confidence interval also showed that the estimates of the patients cured of TB and TB co-infected with HIV were significant. In addition, female TB patients had a higher risk than male patients (Table 6).

The Cox proportional hazard regression model in Table 6 indicates that age significantly affects the survival of TB patients (i.e., it was a risk factor) with a hazard ratio of 1.020326 [95% C.I.: 1.012896–1.02781, p = 0.000]. Meanwhile, gender did not significantly affect the survival of TB patients (i.e., it was not a risk factor) as the hazard ratio is 1.035359 (95% CI: 0.7742109–1.384594, p = 0.815]. From Table 6, the hazard ratios for age and gender are 1.020326 and 1.035359, respectively. As both are greater than 1, the hazard increases with age and gender. Furthermore, the Cox proportional hazard regression model in Table 7 indicates that age and gender did not significantly affect the survival of TB patients co-infected with HIV (i.e., it was not a risk factor) as the hazard ratios are 1.017862 [95% C.I.: 0.9925743–1.043794, p = 0.168] and 0.8985374 [95% C.I.: 0.5210642–1.549463, p = 0.700], respectively. The hazard ratio for age (>1) indicates that the hazard increases with age, while that for gender (<1) indicates that male patients have a lower risk than female patients (Table 7).

## 6. Conclusion and recommendations

In this study, the bounded cumulative hazard function model was extended to accommodate infectious diseases with co-infections by deriving an appropriate probability density function, determining the distribution, and using real data. A cure rate parameter was estimated, and the risk factors were identified. The cure status of TB patients and TB patients co-infected with HIV at the UITH, Nigeria from 2000 to 2015 was considered. The survival time of the patients was estimated using the Kaplan-Meier method. Using the Cox proportional hazard model, covariates that significantly influence the survival of the patients were identified (i.e., risk factors). One of the covariates that significantly affected patient survival at the 0.05 confidence level was the age of the TB patients. The proposed model is particularly useful for estimating the cure rate in a hospital setting or the prevalence of diseases in cross-sectional data. As hazard increases with age based on the data used, early screening of patients is highly encouraged. In this study, the dangers that infected patients pose to the society if they do not show up for treatment and if the infection is not detected early are revealed. Therefore, governments and related health authorities are encouraged to take appropriate actions to combat infectious diseases with possible co-infections.

## Supporting information

S1 Data (XLSX)
